# Isochromosome 12p Formation Regulates Vitamin D Metabolism in Testicular Cancer

**DOI:** 10.3390/nu15102384

**Published:** 2023-05-19

**Authors:** Peter Törzsök, Jasper Van Goubergen, Martin Pichler, Renate Pichler, Frédéric R. Santer

**Affiliations:** 1 Department of Urology and Andrology, Paracelsus Medical University Salzburg, 5020 Salzburg, Austria; p.toerzsoek@salk.at; 2 Division of Experimental Urology, Department of Urology, Medical University of Innsbruck, 6020 Innsbruck, Austria; jasper.van-goubergen@i-med.ac.at; 3 Division of Oncology, Department of Internal Medicine, Medical University of Graz, 8010 Graz, Austria; martin.pichler@medunigraz.at; 4 Translational Oncology, University Hospital of Augsburg, 86156 Augsburg, Germany; 5 Department of Urology, Comprehensive Cancer Center Innsbruck, Medical University of Innsbruck, 6020 Innsbruck, Austria

**Keywords:** testicular cancer, isochromosome 12p, Vitamin D, incidence, carcinogenesis, microlithiasis

## Abstract

Isochromosome 12p (iChr12p) is typical in almost all invasive testicular cancers. Increased copy number of genes on 12p is associated with the development of a clinically manifest tumor; however, the causative genes have not yet been identified. Chromosome 12 harbors many genes involved in Vitamin D metabolism. RNAseq analysis of Vitamin D receptor (VDR) genes from the TCGA cohort revealed that clustering of VDR expression signatures could differentiate between pure seminomas and non-seminomatous germ cell tumors (NSGCT). Using TCGA mRNA expression of anabolic (CYP2R1, CYP27A1 and CYP27B1) and catabolic (CYP24A1) Vitamin D enzymes, positive (PTHLH, IFNG, and TNF) and negative (FGF23) feedback regulators could also clearly distinguish between pure seminomas and NSGCT. We hypothesize that the regulation of Vitamin D metabolism might be disturbed through iChr12p formation, influencing testicular carcinogenesis via increased FGF23 and PTHLH expression. While FGF23 represses CYP27B1 and activates catabolism of active hormone, increased PTHLH secretion can lead to hypercalcemia via inactivation of VDR. In conclusion, testicular cancer is associated with extensive modifications in intratesticular Vitamin D homeostasis. Further research is needed to clarify whether Vitamin D deficiency causes the formation of iChr12p and whether Vitamin D deficiency via iChr12p genomic aberration is involved in testicular carcinogenesis.

## 1. Introduction

Testicular germ cell tumor (TGCT) is the most commonly developed cancer among men aged between 15 and 40 years, accounting for 5% of urological tumors, with 3 to 10 cases per 100,000 males/per year recorded in Western countries [1]. Epidemiological risk factors for the development of TGCT include testicular dysgenesis syndrome, hypospadias, decreased spermatogenesis and impaired fertility or disorders of sex development, family history of TGCT among first-degree relatives and the presence of a contralateral TGCT [2,3,4,5,6]. An isochromosome of the short arm of chromosome 12 (iChr12p) is a well-known genetic marker in most invasive TGCT but is not detected in germ cell neoplasia in situ (GCNIS) [7,8,9,10].

Incidence of testicular germ cell tumor increased in recent decades, especially in industrialized countries [11]. Geographic disparities in the incidence of TGCT, with the highest rate in Northern Europe, are well known but lack clear explanations [12]. Understanding the causes of these specific geographic differences in TGCT incidence rates between and within countries would help further our understanding of the aetiology of this cancer entity. Seasonal variation in cancer incidence was previously described for melanoma, prostate cancer and thyroid cancer based on research released by the Swedish Cancer Registry [13]. We recently observed that the registered incidence of TGCT in Austria also has a strong seasonal pattern, with a reduction in the tumor incidence during the summer months and an increase during the winter months [14]. We hypothesize that these temporal patterns may reflect complex biological processes affecting the likelihood of TGCT carcinogenesis. Seasonal patterns may be caused by periods of insufficient Vitamin D supply related to decreased exposure to sunlight during the winter months. However, no research on a possible association between Vitamin D metabolism in the pathogenesis of TGCT and the observed seasonal occurrence is currently available.

1,25-Dihydroxyvitamin D_3_ (1,25(OH)_2_D_3_) is the most active hormonal form of Vitamin D and exerts its action via a specific intracellular Vitamin D receptor (VDR, located on chromosome (chr) 12q12-q14). The VDR regulates the transcriptional activity of 1,25(OH)_2_D_3_-influenced genes through heterodimerizing with other nuclear hormone receptors, in particular the retinoid acid receptor RXRα. Vitamin D-responsive elements (VDREs) localized in the promoter region of DNA of target genes are bound through the VDR-complexes and regulate transcription of target genes [15,16]. The influence of Vitamin D on male fertility, in particular on sperm motility, is well documented and suggests an organ-specific regulation of Vitamin D_3_ metabolism and action in the testes [17].

**Figure 1:**
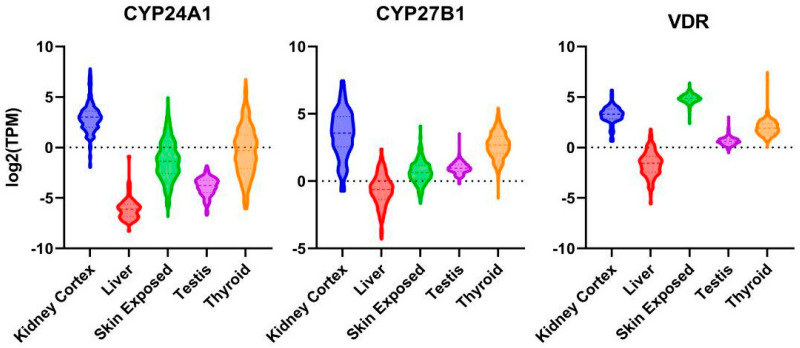
mRNA expression of VDR, CYP24A1 and CYP27B1 in kidney cortex, liver, sun-exposed skin, thyroid gland and testis. Log2 transformed violin plots (expressed in transcripts/million).

Pre-Vitamin D_3_ is produced from 7-dehydrocholesterol in the epidermis of the skin upon UVB exposure via sunlight and isomerizes to Vitamin D_3_. It underlies strong seasonal variations at temperate latitudes, which can be compensated for through dietary intake of Vitamin D_3_. Conversion of Vitamin D_3_ from skin or oral ingestion to the major circulating form 25-hydroxyvitamin D_3_ (25(OH)D_3_) occurs mainly in the liver via CYP2R1, although other organs also express this 25-hydroxylase. Further conversion to 1,25(OH)_2_D_3_ via CYP27B1 (chr12q13.3-q14) mainly occurs in the kidney, contributing to the majority of the circulating 1,25(OH)_2_D_3_-levels; however, numerous organs also express this enzyme, leading to local higher expression levels of the hormone [18]. 25(OH)D_3_ and, preferentially, 1,25(OH)_2_D_3_ can be 24-hydroxylated via CYP24A1, which is the first step in catabolism. Notably, expression of both enzymes, i.e., CYP27B1 and CYP24A1, as well as VDR, are documented in different organs of the male reproductive tract, including the testis [19], Figure 1. 

**Figure 2:**
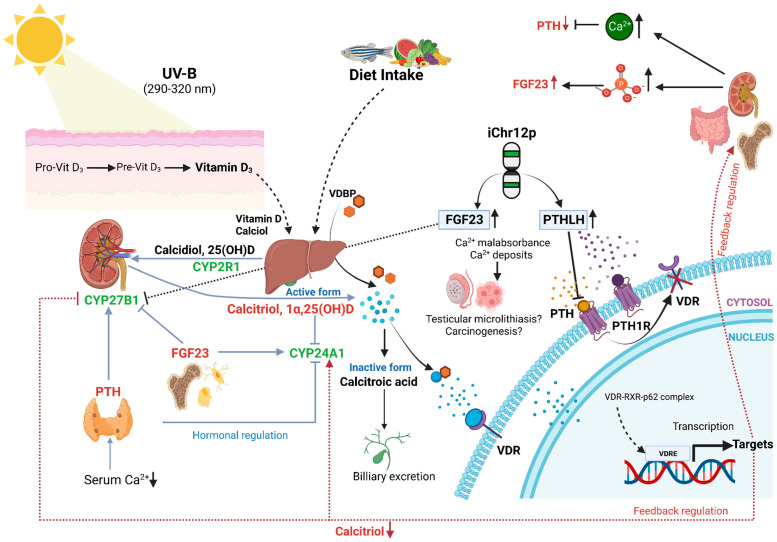
Hormonal and feedback regulation of Vitamin D and possible disturbance through iChr12p formation. Copy number loss of CYP27B1 and VDR, and a copy number gain of FGF23 and PTHLH, are the consequences of iChr12p formation. Consecutively high FGF23 represses CYP27B1 and activates catabolism of active hormone. PTH and PTHLH share same receptor PTH1R. Thus, high PTHLH inhibits binding of PTH to PTH1R. PTHLH is less likely than PTH to stimulate 1,25-dihydroxyvitamin D production and lacks feedback inhibition from plasma calcium. As a result, VDR will be inactivated, and mal-/non-absorbance of calcium leads to calcium deposits. Figure is adapted from [21].

A variety of hormones regulate Vitamin D metabolism [20,21]. Firstly, 1,25(OH_2_)D_3_ regulates its own concentration levels via feedback mechanisms. 1,25(OH_2_)D_3_-activated VDR induces CYP24A1 expression, leading to catabolism of the hormone. The wide expression of CYP24A1 in various organs protects the body against excess concentrations of 1,25(OH_2_)D_3_. On the other hand, 1,25(OH_2_)D_3_ concentration-dependent modulation of CYP27B1 activity is likely to be limited to the kidney. In addition to these feedback regulations, two other hormones, i.e., parathyroid hormone (PTH) and fibroblast growth factor-23 (FGF23, chr12p13.3), both of which play major roles in maintaining calcium and phosphate homeostasis, can regulate Vitamin D metabolism [20]. PTH is secreted through the parathyroid gland when serum calcium levels are low. As a consequence, PTH induces renal expression of CYP27B1, resulting in increased 1,25(OH_2_)D_3_ production. In a feedback mechanism, PTH production is inhibited through high 1,25(OH_2_)D_3_ and calcium levels. FGF23 is secreted through osteocytes and osteoblasts, being able to decrease receptors (FGFR1/3) and the Klotho (KL) co-receptor serum 1,25(OH_2_)D_3_ levels via the FGF through inhibiting the expression of CYP27B1, while stimulating the expression of CYP24A1 in the kidney (Figure 2). In the murine testis, a strong regulation of CYP27B1 expression via FGF23 was documented [22,23]. Cytokines, in particular interferon-γ (IFNG, chr12q14) and tumor necrosis factor-α (TNF, whose receptor TNFRSF1A is located on chr12p13.2), are also strong stimulators of 1,25(OH_2_)D_3_ production in extra-renal tissues [23]. Increased production of PTH-like hormone (PTHLH, a.k.a. PTHRP, Osteostatin, chr12p11.2-p12.1) is a frequent cause of humoral hypercalcemia related to malignancy [24]. PTHLH shares with PTH the same receptor (PTHR1); however, while PTH is a circulating hormone, PTHLH acts in a paracrine or autocrine manner [25]. PTHLH expression is suppressed using 1,25(OH_2_)D_3_ on the transcriptional and post-translational levels [26]. In summary, numerous factors regulating Vitamin D3 metabolism are located on chr12. Copy number amplification of 12p with potential deletion of one copy of 12q via isochromosome formation in testicular carcinogenesis may disrupt this complex regulation of Vitamin D action, as well as calcium and phosphate homeostasis. Disturbance of calcium homeostasis in the testis via isochromosome 12p formation might also be the cause of the frequently observed testicular microlithiasis in TGCT cases [27]. The aim of this study was to evaluate, for the first time, a possible association between Vitamin D in the pathogenesis of TGCT and the presence of iChr12p formation.

## 2. Materials and Methods

### 2.1. Cell Lines and Chemicals

TCam-2, NT2D1, 2102EP, and NCCIT were a kind gift provided by Prof. Hubert Schorle (University of Bonn, Bonn, Germany) and were authenticated via short tandem repeat analysis in case STR profiling data was available in public repositories. Cells were grown in RPMI (TCam-2) or DMEM (NT2D1, 2102EP and NCCIT) with 10% (*v*/*v*) fetal bovine serum under standard conditions. 1,25(OH)_2_D_3_ was purchased from Merck Sigma-Aldrich (Vienna, Austria, D1530) and dissolved in ethanol at 10 µM stock concentration. All-trans retinoic acid (ATRA) was purchased from Merck Sigma-Aldrich (R2625) and dissolved in DMSO at 100 mM stock concentration. 

### 2.2. Proliferation Assays

Cells were seeded at 30% confluency in 96-wells under either steroid-free conditions (using 10% (*v*/*v*) charcoal-stripped fetal bovine serum) or full growth medium (using 10% fetal bovine serum) and grown for 48 h prior treatment, with increasing concentrations of 1,25(OH)_2_D_3_ and in the absence or presence of 100 nM ATRA. Either 24 or 72 h after hormonal treatment, 1/10 volume WST-1 reagent (Roche, Vienna, Austria) was added to the culture medium, and absorption at 450 nm (with subtraction of the absorption reference values at 650 nm) was measured 150 min after incubation at 37 °C in a BioTek Cytation 5 (Agilent Technologies, Vienna, Austria) plate reader.

### 2.3. Data from GENIE and GTex Repositories

The GENIE cohort v13.0-public [28] was accessed via the cbioportal.org platform [29], and datasets of male GCT were retrieved. The copy number segment file was downloaded and displayed in the IGV browser [30]. The CNV Summary with thresholds set at >0.5 (log2(tumor copy number/normal copy number) for amplifications and <−0.5 for deletions was used to assess the percentage of patients with copy number aberrations. Normalized tissue expression data in transcripts/million were downloaded from the GTex portal and visualized with violin plots generated using GraphPad Prism 9. 

### 2.4. VDR Gene Signature, Clustering Analysis, and Idiogram

The VDR gene signature was curated from the following gene sets available at the Molecular Signatures Database (MSigDB, Broad Institute): STAMBOLSKY_RESPONSE_TO_VITAMIN_D3_UP; non-genomic actions of 1,25 dihydroxyvitamin D3; Vitamin D (calciferol) metabolism; Vitamin D in inflammatory diseases; Vitamin D Metabolism; Vitamin D Receptor Pathway; Vitamin D-sensitive calcium signaling in depression; and from the literature [31]. The full gene list of the VDR gene signature can be found in Supplementary Table S1. The RNA-seq of TCGA PanCancer Altlas collection [32] under the form of Z-scores normalized to diploid samples were downloaded from the cBioPortal platform [29]. Heatmaps were generated via package pheatmap using the Pearson clustering algorithm [33]. Genes from this VDR gene signature located on chromosome 12 were used to create an idiogram using the package CopyNumberPlots [34].

## 3. Results

### 3.1. Chromosome 12p Amplifications in TGCT

**Figure 3:**
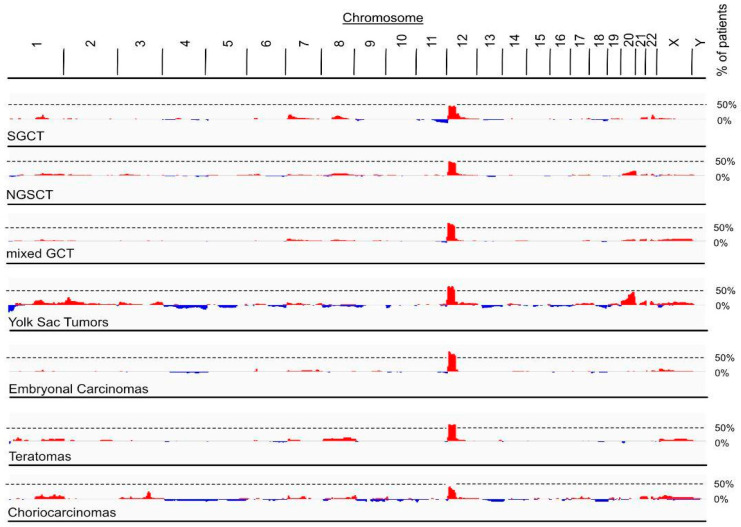
Amplification of chromosome 12p in patients from Genie cohort. Frequency of CNAs in tumor genomes of GCTs retrieved from Genie cohort. Maximal frequency of patients with 12p amplifications was 47% for seminomatous GCT (SGCT, 736 patients, 771 samples), 50% for non-seminomatous GCT (NSGCT, 79 patients, 82 samples), 63% for mixed GCT (339 patients, 350 samples), 65% for yolk Sac tumors (65 patients, 67 samples), 71% for embryonal carcinoma (49 patients, 50 samples), 60% for teratomas (42 patients, 43 samples) and 44% for choriocarcinomas (28 patients, 30 samples). Red, amplifications (log2(tumor copy number/2) > 0.5); blue, deletions (log2(tumor copy number/2) < −0.5.

All invasive TGCT, pure seminomas and non-seminomatous germ cell tumors (NSGCT), and GCNIS cells are aneuploid. The only consistent chromosomal abnormality in invasive TGCT are gains of the short arm of chromosome 12, which are reported to arise via isochromosome formation [35] or reciprocal loss of heterozygosity [36]. Thus, Chr12p copy number alterations (CNA) is a common feature in the majority of patients with invasive TGCT [37]. To assess whether the frequency of chromosome 12p amplifications differentiates between the various histological subtypes of TGCT, we screened CNA data of 12p in 776 patients (801 samples) from the Genie project (Figure 3). Interestingly, pure seminomas and choriocarcinomas presented with the lowest maximal frequency (47% and 44%, respectively) of all GCT histology subtypes, as defined by the Genie project. Higher maximal frequencies were found in NSGCTs (50%), mixed GCT (63%), yolk sac tumors (65%), embryonal carcinomas (71%) and teratomas (60%). Yolk sac tumors also have a high maximal frequency of chromosome 20 amplifications (46%). Therefore, an increase in the copy number of genes on 12p is linked to the development of a clinically manifest TGCT. However, identification of causative genes was hampered by the fact that most 12p gains involve rather large genomic intervals containing unmanageable numbers of candidate genes. Various genes on 12p that could be implicated in testicular carcinogenesis are shown in Table 1, with KRAS being the most frequently mutated gene in TGCT. However, a definite causative agent for chromosome 12p amplifications in TGCT and/or a potential benefit for tumor growth from increasingly expressed genes located on chromosome 12p are currently unknown.

### 3.2. Vitamin D-Responsive Genes on Chromosome 12

**Figure 4:**
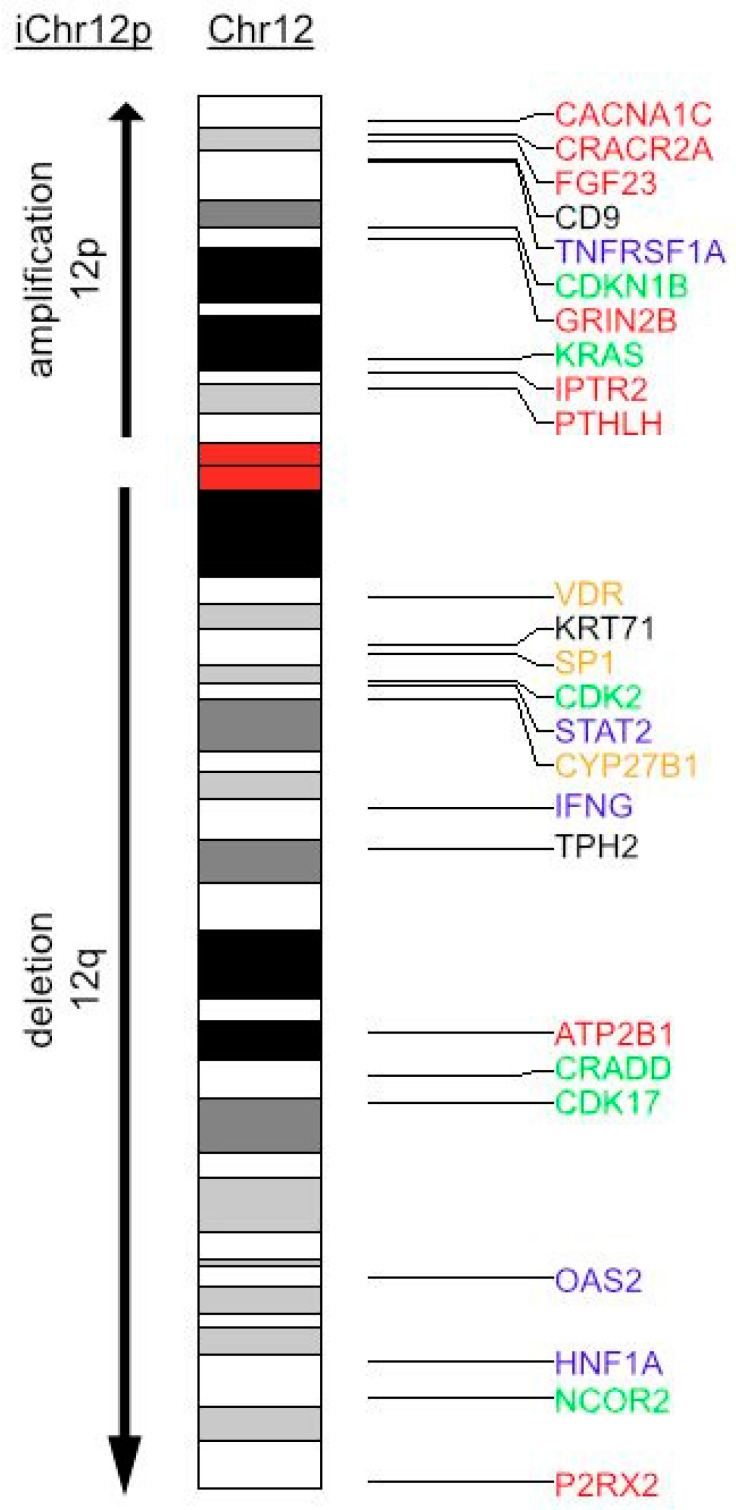
Idiogram of chromosome 12 with annotated VDR gene signature. Genes from VDR gene signature located on chromosome 12 are shown and color-encoded according to their primarily involved signaling pathways. Red: calcium homeostasis; blue: cytokine signaling; green: cell cycle and apoptosis; yellow: Vitamin D metabolism and signaling. During iChr12p formation, an amplification of 12p-arm and a deletion of 12q arm occurs.

Based on the observation of a strong seasonal incidence of TGCT, we hypothesized that the recurrent chromosome 12p amplification (and deletion of chromosome 12q in case of isochromosome formation) in TGCT could involve Vitamin D metabolism and/or action. Indeed, the clinical disorder of Vitamin D dependency rickets type II maps on 12q. Sp1 transcription factor gene (SP1) and VDR are also co-localized on human chromosome arm 12q [38]. To further corroborate an involvement of genes implicated in Vitamin D3 metabolism and action and located on chromosome 12, we used a combined VDR gene signature set (see Section 2). An idiogram of chromosome 12 for genes from this set is shown in Figure 4. From 386 genes in this set, 10 (3.1%) genes localized to chromosome 12p and 15 (3.89%) genes localized to 12q. Those genes were functionally grouped into calcium and phosphate homeostasis, cytokine signaling, cell cycle and apoptosis and Vitamin D metabolism and signaling. Among the calcium and phosphate homeostasis group, important regulators of Vitamin D_3_ metabolism can be found. On 12p, tumor PTHLH secretion can lead to hypercalcemia. FGF23 on 12p represses the 12q-localized CYP27B1 transcription in the kidney and in extra-renal tissues [22]. Further genes regulating Vitamin D_3_ synthesis from the cytokine signaling group are IFNG (12q), as well as possible modified signaling of TNF by a member of the TNF receptor superfamily, i.e., TNFRSF1A (12p). Thus, we hypothesize that Chr12p can formation leads to a copy number gain in FGF23 and PTHLH, and in case of isochromosome formation, to a copy number loss in CYP27B1 and VDR. This finding indicates that disturbances in the feedback loops in Vitamin D metabolism may occur through copy number aberrations.

### 3.3. Expression of Vitamin D-Responsive Genes in TGCT

**Figure 5:**
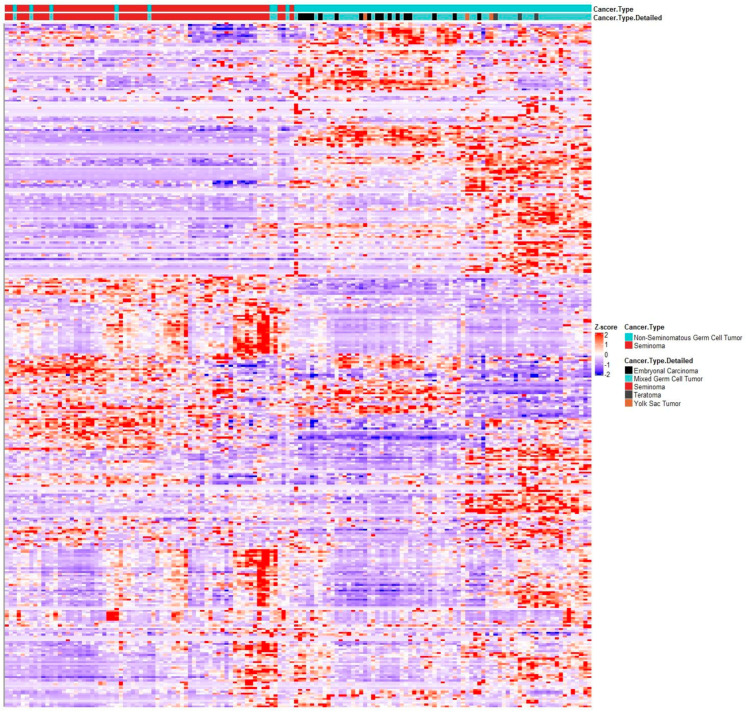
RNAseq clustering analysis of VDR gene signature mRNA expression of genes from VDR gene signature in TGCT dataset from TCGA (144 patients, 60 pure seminomas (in red), 84 NSGCTs (in blue)). NSGCT subtypes are shown (black, embryonal carcinoma; light blue, mixed GCT; grey, teratoma; brown, yolk sac tumor). Heatmap generated via R package heatmap using correlation Pearson clustering algorithm.

To further corroborate a disturbance of Vitamin D metabolism and its consequent genomic and non-genomic action through 12p amplification, we used a publicly available dataset of 144 TGCT patients (60 pure seminoma and 84 NSGCTs) from The Cancer Genome Atlas (TCGA) project encompassing DNA and RNAseq data, as well as clinical annotations [31]. With the help of cbioportal.org, we analyzed mRNA expression of the previously used Vitamin D gene signature. Clustering analyses of these VDR expression signatures were clearly able to discriminate pure seminomas from NSGCT. This signature also showed a tendency to cluster the various NSGCT histology subtypes (Figure 5). The frequency of genomic alterations in genes in the VDR gene signature between seminomas and NSGCTs was comparable: in total, 70% and 63.1% of seminomas and NSGCTs, respectively, had a least one alteration in this gene set. Of these, 35% and 13.1% were mutations, 0% and 1.19% were structural variants, 8.33% and 15.48% were CNAs (amplification or deletion) and 18.33% and 17.86% were multiple alterations. Interestingly, VDR gene expression alone was not able to discriminate between pure seminoma and NSGCT types, leading to the conclusion that its expression and, especially, its activity are likely regulated at the post-transcriptional and/or endocrine level. Unfortunately, exact diagnosis dates are not annotated in the TCGA database, which prevented us from analyzing seasonal variations in the VDR gene signature. From this data, we concluded that VDR and/or its ligand 1,25(OH)_2_D_3_ may have a role in carcinogenesis and/or progression of TGCT and that differential activities, i.e., differential transcriptional programs of the VDR and non-genomic actions correlate with the histology subtype of GCT. Thus, this VDR gene signature may assist histopathological diagnosis of TGCT subtypes.

### 3.4. Vitamin D Response of TGCT Cell Lines on Tumor Cell Viability

**Figure 6:**
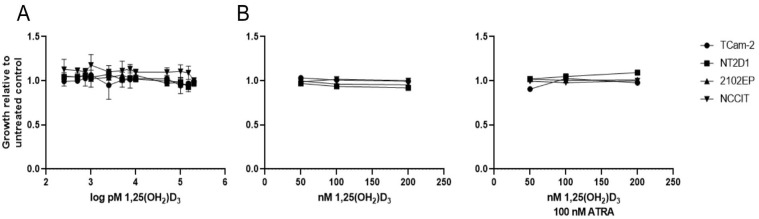
Stimulation with 1,25(OH_2_)D_3_ in seminomatous (TCam-2) and non-seminomatous (NT2D1, 2102EP, NCCIT) TGCT cell lines did not result in reduced proliferation. (**A**) TCam-2, NT2D1, 2102EP and NCCIT cells were treated with increasing concentrations of 1,25(OH)_2_D_3_ (250 pM–200 nM) in steroid-free growth medium for 72h. Error bars showing standard deviation. Experiment was performed at *n* = 3. Number of replicates: 1. (**B**) TCam-2, NT2D1, 2102EP and NCCIT cells were treated with increasing concentrations of 1,25(OH)_2_D_3_ (50–200 nM) in normal growth medium for 24 h in absence (left panel) or presence (right panel) of 100 nM ATRA. (**A**,**B**) Cell growth was assessed with WST-1 assay.

Based on the result that differential transcriptional programs of the VDR and non-genomic actions of Vitamin D3 correlate with the histology subtype, we tested whether Vitamin D3 treatment may affect the proliferative capacity of seminoma and NSGCT cell lines. To this end, we measured proliferation of the one available pure seminoma (TCam-2) and three NSGCT (NT2D1, NCCIT and 2102EP) cell lines via WST-1 assay after treatment with different concentrations of 1,25(OH)_2_D_3_. The median concentration of 1,25(OH)_2_D_3_ in the body is 59.4 pg/mL (142 pmol/L) [39], which has a short half-life of a few hours in serum [40]. For this reason, we tested over a wide concentration range (50 pmol/L–200 nmol/L) using charcoal-stripped serum in order to remove steroids from the culture medium that might interfere with Vitamin D action. However, in contrast to a previous study [31], we were not able to find an effect on proliferation on either cell line (Figure 6A). To assess whether co-activation of the VDR’s dimerization partner RXR is needed to influence proliferation of these cell lines, co-treatment with all trans retinoic acid (ATRA, 100 nM) and 1,25(OH)_2_D_3_ was performed. Moreover, after a shorter incubation time, growth in non-steroid depleted medium and addition of all trans retinoic acid (ATRA, 100 nM), the ligand of RXR did not result in reduced proliferation in either cell line (Figure 6B). We, therefore, concluded that Vitamin D has no effect on proliferation of seminomatous and non-seminomatous cell lines, albeit an active VDR gene signature is present. 

### 3.5. Expression of Regulators of Vitamin D Metabolism in TGCT

**Figure 7:**
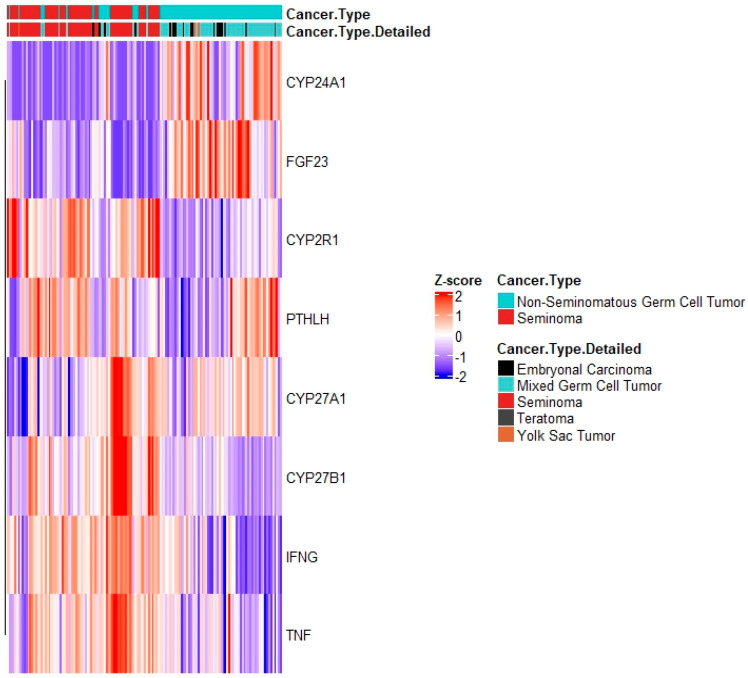
mRNA expression of vitamin D regulatory genes in TCGA’s TGCT dataset. Analysis comprises anabolic (CYP2R1, CYP27A1 and CYP27B1) and catabolic (CYP24A1) enzymes, as well as positive (PTHLH, IFNG, and TNF) and negative (FGF23) feedback regulators. Heatmap generated via R package heatmap using correlation Pearson clustering algorithm.

To discover whether differences in local concentrations of Vitamin D in the carcinogenic areas of the testicle might be responsible for the different VDR gene signatures between the histology subtypes, we assessed the expression of key regulators of Vitamin D metabolism in the TCGA dataset. This method included the anabolic (CYP2R1, CYP27A1 and CYP27B1) and catabolic (CYP24A1) enzymes, as well as positive (PTHLH, IFNG, and TNF) and negative (FGF23) feedback regulators. High mRNA expression of anabolic regulators and enzymes of Vitamin D was found in pure seminomas, while catabolic regulators and negative feedback enzymes are highly expressed in NSGCT (Figure 7). This shows that the differential activation of VDR signatures genes in seminomas and NSGCTs might be the result of GCT subtype-specific concentrations of 1,25(OH_2_)D_3_. These differences in local 1,25(OH_2_)D_3_ concentrations might be a cause of differential expression and activity of Vitamin D regulators mediated via chromosome 12 CNAs. In situ determinations of 1,25(OH2)D_3_ concentrations in malignant areas of both GCT subtypes and correlation with expression and activity of Vitamin D regulators, along with determination of chromosome 12 CNAs, may confirm this finding.

## 4. Discussion

The VDR is expressed in over 30 human tissues involved in the reproductive system, such as testis, uterus, and ovary, and it was also previously found in the immune system. Expression of VDR was shown to exhibit tumor-suppressing and anti-proliferative effects, promoting apoptosis and inhibiting angiogenesis [41]. Extensive research suggested that Vitamin D deficiency is associated with the increased risk of cancer [42]. The over-expression of VDR is described as an endogenous reaction to tumor progression correlated with a favorable prognosis [43,44]. A high expression of VDR is strongly linked to a lower chance of prostate cancer progression and cancer-related death [43]. Thus, we hypothesize that seasonality of testicular cancer incidence might be influenced through Vitamin D supporting the relationship between Vitamin D and cancer. Several epidemiologic trials confirmed that a low Vitamin D status is also a risk factor for several types of cancer, such as breast cancer [45], prostate cancer [46,47] and colorectal cancer [48], suggesting that Vitamin D supplementation might be cancer preventive. Nevertheless, there are still conflicting data in this field, and large randomized control trials could not corroborate these findings for the general population [49]. Moreover, current evidence from cell experiments, ecological and observational studies and RCTs does not allow us to give definitive answers regarding whether Vitamin D supplementation can prevent and treat cancer [50]. A possible explanation might be that accumulating data suggest that the metabolism and functions of Vitamin D are dysregulated in many types of cancer, conferring resistance to the antitumorigenic effects of Vitamin D supplementation and thereby contributing to the development and progression of cancer [21]. This fact might also explain our findings showing no significant decrease in proliferation in four different testicular cancer cell lines stimulated with 1,25(OH)_2_D_3_.

Vitamin D metabolism is active in normal testes but is lost during the malignant progression from GCNIS to overt TGCT. TGCT is associated with extensive modifications in intratesticular Vitamin D, calcium and phosphate homeostasis [51]. In TGCT, it was demonstrated that Vitamin D also regulates testicular cell proliferation and apoptosis [31,52]. Initial evidence of testicular expression of VDR was also reported in vitro and in vivo [31]. Another in vitro study of VDR expression confirmed high nuclear and cytoplasmic staining in an embryonal carcinoma cell line, where VDR was inducible with the addition of Vitamin D but downregulated through testosterone [53]. A specific correlation was also found between VDR, enzymes involved in Vitamin D metabolism and cancer cell differentiation: high levels are observed in carcinoma in situ, whereas low levels are found in invasive seminoma [54]. In addition, Vitamin D3 deficiency was associated with embryonal carcinoma and advanced clinical tumor stage in testicular cancer [55]. Interestingly, a high prevalence of Vitamin D_3_ deficiency was confirmed in TGCT up to 83% [55]. In contrast, no permanent reduction in serum 25(OH)D_3_ levels after orchiectomy but a transient post-operative drop of 25(OH)D levels were found in 177 TGCT patients [56]. Nevertheless, a recent study by Lesko et al. demonstrated that low-plasma Vitamin D was associated with an unfavorable response to therapy and disease recurrence in TGCT [57]. 

In addition, VDR expression and individual enzymes playing a role in Vitamin D metabolization in human spermatozoa can differentiate between normospermic patients and men with subfertility [19,58,59]. In an animal study with VDR-null mice and rodents, Vitamin D deficiency showed an association with decreased fertility because of impaired sperm production and bad sperm motility [54]. Vitamin D may play a role in metabolism during fetal development, as VDR expression was demonstrated in fetal gonocytes and pre-spermatogonia [59]. The expression of VDR in Sertoli cells is controlled through follicle-stimulating hormone (FSH), where it plays a role in germ cells’ maturation and motility [19]. Cellular investigations demonstrated that Vitamin D impacts the activity of various immune cells, such as epithelial cells, macrophages, monocytes and activated CD4 T cells [60,61,62]. Vitamin D_3_ can induce apoptosis and cell cycle arrest in endothelial cells isolated from tumor, while this effect was not observed in cases of normal endothelial cells [63]. TGCT patients were revealed to have low levels of Vitamin D on long-term follow-up [64]. The lack of Vitamin D_3_ is also associated with erectile dysfunction, lower sperm motility and decreased sperm concentration, emphasizing its role in male infertility [19,65]. In a recent study, idiopathic male infertility was associated with Vitamin D_3_ deficiency [66]. Vitamin D_3_ can significantly reduce oxidative stress on aged rat testis through the antioxidant defense system [52]. In addition, humans who took daily doses of vitamin D showed decreased oxidative DNA damage [67]. Oxidative stress was shown to be associated with male infertility [68]. Higher levels of oxidative stress were seen in patients with reduced sperm counts and concentrations [69]. The testicles with normal spermatogenesis demonstrated higher VDR expression on IHC when compared with biopsies from patients with hypospermatogenesis [53].

Although testicular microlithiasis is more common in TGCT patients, it is not an independent risk factor for testicular cancer [70]. As we previously mentioned, iChr12p formation might lead to a copy number loss of CYP27B1 and possibly VDR, as well as a copy number gain of FGF23 and PTHLH. In detail, high FGF23 represses CYP27B1 and activates the catabolism of active hormones. Blomberg et al. demonstrated an overexpression of FGF23 in GCNIS contributing to the formation of testicular microlithiasis [51]. High PTHLH may compete with parathyroid hormone (PTH) for binding to parathyroid hormone 1 receptor (PTH1R), thus leading to inactivation of VDR activity and mal- or non-absorbance of calcium, leading to calcium deposits, as shown in Figure 2. These increased calcium deposits may explain the presence of testicular microlithiasis, which is a condition where calcium deposits form in the lumina of seminiferous tubules or arise from the tubular basement membrane components [71]. Underlining the possible association between vitamin D metabolism and iChr12p formation, previous immuno-histochemistry (IHC) analysis showed that VDR expression was significantly lower in pure seminoma, and not present in GCNIS, compared to NSGCT [31]. In line with these findings, iChr12p was not detected in GCNIS and pure seminomas appear to lack a gain of 12p [7,8,9,10]. In our analysis, the highest frequency of iChr12p amplification was shown in NSGCTs, such as yolk sac tumors (65%), embryonal carcinomas (71%), etc. Furthermore, there was no difference in VDR expression on IHC between metastatic and non-metastatic TGCTs [64].

Metabolism of Vitamin D appears to be restored in the process of tumor differentiation, as it was observed in the differentiated somatic and extraembryonic components of teratomas [72]. A clear decrease in VDR and Vitamin D-metabolizing enzyme expression was seen in invasive TGCTs when compared to pre-invasive GCNIS. As opposed to GCNIS, expression of VDR and Vitamin D-metabolizing enzymes was non-existent or low for embryonal carcinoma (EC) cells, implying that this pathway was deactivated during the shift from GCNIS to EC [59]. Numerous genes involved in Vitamin D metabolism are located on chromosome 12, which might be disturbed through iChr12p formation. According to our data, TCGA mRNA expression of vitamin D regulatory genes can accurately differentiate between pure seminomas and NSGCT. CYP24A1, i.e., one of the Vitamin D regulatory genes, which can help to distinguish between pure seminomas and NSGCT, can serve as a prognostic tool to predict good sperm quality and, thus, fertility [19,72]. Furthermore, the proportion of sperm with CYP24A1 expression in a prospective study was revealed to be a better marker of successful pregnancy outcome after intrauterine inseminations than sperm motility and concentration [73]. The oncogenic role of CYP24A1 was also shown in breast, prostate, and lung cancer [21]. Its inhibition results in a higher local concentration of calcitriol, thus leading to an enhanced antitumor effect [74]. It was previously suggested that the overexpression of CYP24A1, i.e., inactivation of calcitriol, could be critical for progression of colorectal adenoma to carcinoma [75].

## 5. Conclusions

iChr12p is a common feature in patients with invasive testicular cancer. However, candidate genes involved in the mechanism that gain 12p sequences and, thus, in testicular carcinogenesis have not been identified to date. Importantly, chromosome 12 harbors many genes that are involved in cellular calcium homeostasis and Vitamin D metabolism. RNAseq analysis of VDR genes and TCGA mRNA expression of Vitamin D regulatory genes were clearly able to discriminate pure seminomas from NSGCT. We hypothesize that the regulation of Vitamin D metabolism might be disturbed through iChr12p formation influencing testicular carcinogenesis. Further prospective research is now needed to determine which role iChr12p formation and Vitamin D metabolism plays in testicular tumorigenesis, as well as the potential for them to become targets for future therapies or diagnostic tools. Thus, a prospective study is planned to correlate iChr12p formation with altered VDR gene signature in seminoma and NSGT via RNAseq. Although our data do not provide proof that seasonal variations impact TGCT carcinogenesis, in situ determinations of Vitamin D concentrations and correlation with iChr12p formation in TCGT patients are planned, correlating iChr12p formation with the diagnosis date.

## Figures and Tables

**Table 1:** Amplified genes on 12p that could be implicated in testicular carcinogenesis. OnkoKB^TM^ cancer genes with copy number aberrations (amplifications) localized on chromosome 12p retrieved from Genie cohort of male GCTs with following histology subtypes: mixed GCT, seminoma, NSGCT, yolk sac tumor, embryonal carcinoma, all teratomas and choriocarcinoma. Total profiled samples: 702. Number of samples with CNA, number of total samples profiled, and resulting percentage are reported.

Gene	Description	Cytoband	Samples with CNA	Samples Profiled	Frequency of CNA
CCND2	Cyclin D2, cell cycle regulator	12p13.32	142	701	20.3%
CDKN1B	p27^KIP1^, inhibitor of cyclin D-CDK4 complexes	12p13.1	163	701	23.3%
CHD4	Helicase, epigenetic transcriptional repression	12p13.31	1	5	20.0%
ETV6	ETS family transcription factor	12p13.2	134	698	19.2%
FGF23	Growth factor, phosphate homeostasis	12p13.32	1	6	16.7%
FGF6	Growth factor controlling cell proliferation/differentiation	12p13.32	1	6	16.7%
H3F3C	Histone of the H3 family	12p11.21	103	594	17.3%
KDM5A	Demethylase of Lysine 4 of histone H3	12p13.33	122	638	19.1%
KRAS	Proto-oncogene, small GTPase	12p12.1	135	702	19.2%
PIK3C2G	Member of the phosphoinositide 3-kinase family	12p12.3	102	599	17.0%
PTPN6	Protein tyrosine phosphatase	12p13.31	1	1	100.0%
PTPRO	Receptor-type protein tyrosine phosphatase	12p12.3|12p13-p12	1	1	100.0%
RAD52	DNA repair	12p13.33	130	637	20.4%
RECQL	Helicase involved in DNA repair	12p12.1	58	300	19.3%

## Data Availability

The data used for the analyses described in this manuscript were obtained from the GTEx Portal on 04/01/23.

## Supplementary Materials

The following supporting information can be downloaded at: www.mdpi.com/article/10.3390/nu15102384/s1, Table S1: List of the VDR gene signature.
